# Perioperative morbidity and mortality in octogenarians sustaining traumatic osteoporotic type 4 and 5 thoracolumbar and lumbar fractures: a retrospective study with 3 years follow-up

**DOI:** 10.1007/s00701-023-05564-z

**Published:** 2023-04-13

**Authors:** Pavlina Lenga, Gelo Gülec, Karl Kiening, Andreas W. Unterberg, Basem Ishak

**Affiliations:** Department of Neurosurgery, Heidelberg University Hospital, Im Neuenheimer Feld 400, 69120 Heidelberg, Germany

**Keywords:** Thoracolumbar osteoporotic fractures, Octogenarians, Instrumentation

## Abstract

**Purpose:**

This study aimed to guide the more efficient management of type 4 and 5 thoracolumbar or lumbar osteoporotic fractures (OF) in patients aged 80 years and older with an acute onset of neurological decline. This aim was achieved by assessing the clinical course and morbidity and mortality rates and identifying potential risk factors for patient mortality

**Methods:**

Electronic medical records were retrieved from a single institution pertaining to the period between September 2005 and December 2020. Data on patient demographics, neurological conditions, surgical characteristics, complications, hospital course, and 90-day mortality were also collected.

**Results:**

Over a 16-year period, 35 patients aged ≥80 years diagnosed with thoracolumbar and lumbar OF were enrolled in the study. The mean Charlson comorbidity index (CCI) was >6, indicating a poor baseline reserve (9.4 ± 1.9), while cardiovascular diseases were the most prevalent among comorbidities. The mean surgical duration was 231.6 ± 89.3 min, with a mean blood loss of 694.4± 200.3 mL. The in-hospital was 8.6% and 90-day mortality rates at 11.4%. Two patients underwent revision surgery for deep wound infection. Intraoperative and postoperative radiography and computed tomography (CT) imaging revealed correct screw placement. Proper alignment of the thoracolumbar spine was achieved in all the patients. Unique risk factors for mortality included the presence of comorbidities and the occurrence of postoperative complications.

**Conclusions:**

Emergent instrumentation in patients with acute onset of neurological decline and potentially unstable spines due to thoracolumbar and lumbar OF improved functional outcomes at discharge. Age should not be a determinant of whether to perform surgery.

## Introduction

The geriatric population represents the fastest-growing demographic worldwide, and individuals aged 80 years and older are expected to triple by 2050 [1]. In this aging society, geriatric trauma involving spinal injury has gained increasing attention, posing a unique challenge for a sufficient therapeutic resume. The vulnerability of the geriatric population to spinal cord injury is attributable to a multitude of compounding factors, such as the loss of bone mineral density, increasing degenerative changes of the spinal cord, amenability to fall-related injuries due to a substantial decrease in neurological reserve, and an increased rate of motor vehicle accidents per mile driven [18, 19, 33].

Thoracolumbar osteoporotic fractures (OF) are the most common type of fractures among the elderly [3], resulting in difficulties with activities that are part of their daily routine and conferring a higher risk of hospitalization and mortality. Surgical management with posterior instrumentation, primarily concomitant cement-augmented screws, is recommended for OF cases resulting in deformation with distinct involvement of the posterior wall, as well as in cases presenting with loss of the vertebral frame or vertebral collapse [6]. However, it is important to highlight that increasing age is an independent risk factor for poor outcomes after traumatic injury and a higher risk of death [4, 15]. For instance, underlying illnesses such as renal or hepatic failure, chronic steroid use, or malignancy might increase the mortality risk in elderly patients after trauma by up to five times [4]. In the case of spinal surgery for the management of traumatic vertebral fractures, increasing age and poor neurological baseline status with concomitant comorbidities have been described as putative risk factors contributing to higher rates of perioperative and postoperative complications and mortality [24]. However, a systematic analysis focusing solely on morbidity and mortality rates for OF exclusively in octogenarians is still lacking.

Owing to the lack of robust clinical evidence on this topic, this study sought to provide a guide for more efficient management of thoracolumbar or lumbar OF in patients aged ≥80 years with an acute onset of neurological decline by assessing the clinical course and morbidity and mortality rates and determining potential risk factors for patient mortality.

## Methods

Clinical and imaging data were retrospectively collected between September 2005 and December 2020 from the database of our institution. This study was approved by the local ethics committee of our institution (approval number 880/2021) and conducted in accordance with the Declaration of Helsinki. The requirement for informed consent was waived because of the retrospective nature of the study.

Patients aged ≥80 years with traumatic OF types IV and V according to the recommendations of the Spine Section of the German Society for Orthopaedics and Trauma system (DGOU) diagnosed on computed tomography (CT) images were included [6]. Magnetic resonance imaging (MRI) of the thoracolumbar and lumbar spine was performed to evaluate spinal ligament integrity. The exclusion criteria were as follows: congenital instability, rheumatoid arthritis, instability caused by a tumor, spinal infections, and previous spinal surgery in the fracture region. The German guidelines for trauma mechanisms were used to define low-energy trauma (LET), and patient injuries were classified accordingly [28]. LET was defined as a fall from a sitting or standing position and low height (<1 m) [28].

### Clinical record

Data regarding patient demographics, comorbidities, American Society of Anesthesiologists scores, surgical duration, number of treated spinal levels, perioperative and postoperative complications, length of hospital stay, intensive care unit (ICU) stay, readmission, reoperation, and mortality were retrieved from the patients’ electronic records. Preoperative comorbidities were assessed using the age-adjusted Charlson comorbidity index (CCI). CCI was calculated for each patient and classified as no comorbidity, minimal comorbidity, moderate comorbidity, or severe comorbidity (CCI of 0, 1 or 2, 3 to 5, and >5, respectively) [10, 12]. Pre-treatment neurological condition was assessed using the motor score (MS) of the American Spinal Injury Association impairment grading system (MS = 0, no muscle strength; MS = 100, healthy). Posttreatment MS data were obtained from the last documented clinical encounter. Routine clinical and radiological follow-up examinations were performed before discharge and 3 months after surgery according to our institutional standards. Patients on anticoagulants received antidotes before surgery based on the German Guidelines for interrupting the anticoagulation effects [31]. An experienced anesthesiologist determined the doses of the antidote medication according to current guidelines, which consider the baseline characteristics (such as age, BMI, and renal function) of each patient. None of the patients presented with clinical or laboratory signs of infection before surgery. The follow-up period was 3 and 72 months after surgery. Conventional radiographs in the anteroposterior and lateral views were obtained to evaluate screw position.

### Decision making

**Fig. 1:**
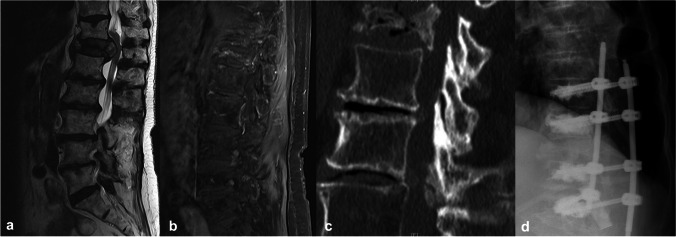
**a** Sagittal T2-weighted and **b** postcontrast magnetic imaging of a traumatic Th 12 fracture of an 85-year-old female patient presenting with progressive motor weakness of the low extremities. **c** Lateral CT scan depicting the osteoporotic Th 12 fracture. **d** Lateral radiographic view of cement-augmented posterior screw fixation extending from level Th10 to L2

Plane dynamic radiography was performed before surgery to evaluate the stability of the fracture. In addition, CT was performed to assess the morphology of the fractures and the presence of osteoporosis [14, 37]. Osteoporotic signs were identified by the degree of vertebral reduction, cortical disruption, and impaction of trabeculae, with increased density adjacent to the endplate. The final decision for augmentation was made by an experienced surgeon based on the mechanical strength of the implanted pedicle screw. Since there is no consensus on when to perform short- or long-segment posterior instrumentation for such fractures, the number of cement-augmented screws varied between four and six cement-augmented pedicle screws (Fig. 1). Type 4 fractures were mainly treated with four cement-augmented pedicle screws, while type 5 fractures were treated with six cement-augmented pedicle screws due to the presence of distraction or rotation. However, the preoperative premorbid status of the patients and the associated risks of longer surgical duration contributed significantly to the decision-making process. After a meticulous interdisciplinary evaluation of both clinical and radiological parameters and weighing the pros and cons of longer surgical times, experienced spine surgeons (BI and KK) decided on the safest surgical procedure that preserved spinal stability and minimized the risk of perioperative and postoperative complications. After surgery, patients were transferred to either the normal ward or intensive care unit, depending on the surgical duration and presence of intraoperative and perioperative complications. Pain medication was administered according to the WHO analgesic ladder [2], and patients received professional physiotherapeutic support for mobilization starting on the first day after surgery. An orthosis was not required as the spinal column was evaluated as stable after surgery.

### Surgical technique

All surgeries were performed by two experienced surgeons [KK and BI] under general anesthesia. Patients were placed in a prone position on a carbon table. First, a midline incision was made to expose bony structures. Four to five navigation reference marker screws (4-mm self-drilling screws; Zimmer Biomet Holdings, Inc., Warsaw, IN, USA) were inserted into the laminae and spinous processes. Subsequently, CT was performed under sterile conditions (Siemens, CT Emotion, Sliding Gantry, Siemens, Erlangen, Germany). The emerging dataset (Stryker Navigation System II with SpineMap^TM^ and NAV3i^TM^ 3D-Navigation, Kalamazoo, Michigan, USA) was used to determine the insertion points, using marker screws as a reference, and to identify the exact position, trajectory length, and diameter of the pedicle screws. The reference clamp was attached, the marker screws were merged with the navigation, and pedicle insertion was performed. A calculated navigation accuracy of 0.3–0.5 mm was deemed appropriate and met in all patients.

Screw placement was performed according to the technique described by Weinstein et al. [35] The screws were fully cannulated polyaxial titanium alloy screws. The radial holes were all within the distal third of the screw (expedium [DepuySynthes] and XIA [Stryker]), varying in length and diameter according to each patient’s individual anatomical characteristics.

The length of screw fixation ranged from 2 to 4 segments and was fixed on the vertical segment that included the fractured part. Type 4 fractures (burst fractures) were mainly treated with four cement-augmented pedicle screws, while type 5 fractures, due to the presence of rotation or distraction, were treated with six cement-augmented pedicle screws. In cases where the vertebral body and pedicle were severely damaged and screw insertion was difficult, screws were not inserted. Fixation was extended to four segments if it was considered difficult to achieve stability with fixation of only the vertical segment due to a damaged posterior column in over two segments. If bone fragments remained in the spinal canal, posterior pedicle fixation was followed by decompression. Autogenous bone was applied for posterolateral fusion. The senior surgeon made the decision to use cement-augmented pedicle screws intraoperatively based on the bone quality.

Under intermittent X-ray fluoroscopy, approximately 2–4 mL of polymethylmethacrylate per vertebral body was administered to the thoracic or lumbar spine. All procedures were performed according to the VERTECEM Bone Cement (DepuySynthes) and CORTOSS (Stryker) guidelines. In cases of cement leakage with X-ray fluoroscopy, a CT scan of the instrumented area was performed to detect the amount of cement leakage in the spinal canal or neural foramina. Cement preparation and application were performed according to the manufacturer’s instructions given by the company. In particular, the 30-s phase of mixing powder and liquid, 30-s application of device filling, and 300-s waiting period had to be maintained before use of the cement, until its consistency was in a tooth-like paste state. The injection was interrupted when cement leakage was observed.

### Statistical analyses

Categorical variables are presented as numbers and percentages. Continuous variables are presented as means ± standard deviations, and the Shapiro–Wilk test was used to verify whether the data distribution was normal. Baseline and surgical characteristics, perioperative and postoperative complications, length of stay (LOS), ICU stay, readmission, reoperation, and mortality were compared group-wise using independent *t*-tests for continuous variables and chi-squared tests for categorical variables. The Wilcoxon rank test was used to evaluate changes in neurological status (MS). In the second-stage analysis, a binary logistic regression analysis was performed to identify the risk factors for mortality. Statistical significance was set at a *p*-value ≤ 0.05.

## Results

Over a period of 15 years, 35 patients aged ≥ 80 years who were diagnosed with thoracolumbar and lumbar OF and underwent CT-based point-to-point navigation were enrolled in this study. The mean age was 82.0 ± 1.2 years, with a predominance of female patients (*n* = 23, 65.7%). The mean CCI was 9.2 ± 2.1, indicating a poor baseline reserve. Cardiovascular disease had the highest prevalence. Most of the OF were located in the thoracolumbar spine (*n*=23, 65.7%). A notable decline was seen in neurological condition and functional status, as defined by MS (mean MS 84.1±12.1). Eleven individuals (31.4%) were taking anticoagulant agents and had a pathologic PTT of 47.6±3.2 s. A detailed description of baseline characteristics is provided in Table 1.

**Table 1:** Baseline patient characteristics

	Decompression *N*^a^ = 35
Age, years (mean, SD^b^)	82.0 (1.2)
Sex (*n*^c^, %)	
Male	12 (34.3)
Female	23 (65.7)
BMI^d^, kg/m^2^ (mean, SD)	24.5 (4.1)
Comorbidities	
Age-adjusted CCI^e^ score (mean, SD)	9.4 (1.9)
Arterial hypertension (*n*, %)	31 (91.5)
Myocardial infarction (*n*, %)	20 (57.1)
Coronary heart disease (*n*, %)	17 (48.6)
Atrial fibrillation (*n*, %)	17 (48.6)
Heart failure (*n*, %)	6 (17.1)
COPD^f^ (*n*, %)	11 (31.4)
Diabetes mellitus type II (*n*, %)	4 (11.4)
Renal failure (*n*, %)	5 (14.3)
Liver disease (*n*, %)	3 (8.6)
Gastrointestinal ulcer (*n*, %)	6 (17.1)
TIA^g^/stroke (*n*, %)	8 (22.9)
Malignancy (*n*, %)	10 (28.6)
Dementia (*n*, %)	3 (8.6)
Previous spinal surgery (*n*, %)	3 (8.6)
ASA^h^ class (*n*, %)	
I	
II	9 (25.7)
III	24 (68.6)
IV	2 (5.7)
Localization (*n*, %)	
Thoracolumbar	23 (65.7)
Lumbar	12 (34.3)
Preoperative MS^i^ score (mean, SD)	84.1 (12.1)

^a^Group size, ^b^standard deviation, ^c^number of patients, ^d^body mass index, ^e^Charlson comorbidity index, ^f^chronic obstructive pulmonary disease, ^g^transient ischemic attack, ^h^American Society of Anesthesiologists, ^i^motor score of the American Spinal Injury Association grading system

### Surgical outcomes

As shown in Table 2, the mean surgical duration was 231.6 ± 89.3 min, with a mean blood loss volume of 694.4 ± 200.3 mL. The mean number of fused levels was 2.4 ± 1.6. The mean ICU stay was 2.4 ± 1.6 days, while the hospital stay lasted 12.6 ± 6.7 days. During the hospital stay, three patients (8.6%) died: two as a result of septic pneumonia and one due to heart failure. The 90-days mortality was 2.9 and was not surgery-related. Two patients underwent revision due to deep wound infection, while no further surgeries were required during the follow-up period due to screw loosening or secondary instability. The MS improved significantly after surgery (preoperative MS 84.1±12.1 vs. postoperative MS 92.8±11.6; *p*<0.001). The mean follow-up period was 27.1 ± 12.1 months. Intraoperative and postoperative radiographic and CT images revealed correct screw placement. Proper alignment of the thoracolumbar spine was achieved in all the patients.

**Table 2:** Surgical characteristics and clinical course

	Decompression *N*^a^ = 35
Surgical duration, min	231.6 (89.3)
No. of levels decompressed/fused	2.4 (1.6)
Blood loss (ml)	694.4 (200.3)
Intraoperative blood transfusion	10 (28.6)
Hospital stay duration, days	12.6 (6.7)
ICU^b^ stay, days	2.4 (1.8)
Mortality	
In-hospital (*n*^c^, %)	3 (8.6)
90-day (*n*, %)	4 (11.4)
Post MS^d^	92.8 (11.6)

Except where otherwise indicated, the quantities are presented as mean (standard deviation, SD)

^*^Significant difference; post, after surgery; ^a^group size, ^b^intensive care unit, ^c^number of patients, ^d^motor score of the American Spinal Injury Association grading system

Thirty patients diagnosed with type 4 and type 5 osteoporotic fractures (OF) were assessed in this study. Of these, 28 patients with type 4 fractures underwent four-segment posterior instrumentation, while two patients from the same group underwent six-segment posterior instrumentation based on intraoperative surgeon decision-making. Patients with type 5 fractures received only six-segment posterior instrumentation. Both procedures resulted in substantial neurological improvement among patients. However, patients with long-segment fixation experienced higher pain levels during postoperative mobilization, as documented by the visual analog scale (VAS) score (4-segment, 6.2±1.5 vs. 6-segment, 7.1±1.1; *p*=0.056). However, this finding did not reach statistical significance.

### Complications

Postoperatively, all patients were admitted to the ICU. Twenty-two patients experienced postoperative complications and were closely monitored for vital parameters and underwent daily laboratory tests. Patients with deep wound infections received a change of wound dressing twice a day by a physician and underwent daily laboratory tests, with a special focus on blood infection levels, to avoid delays in the early diagnosis of sepsis. Six patients were immediately transferred to the ICU after surgery due to intraoperative respiratory and cardiac irregularities. These patients were subsequently diagnosed with pneumonia (*n*=3) and acute heart failure (*n*=3). Revision surgery was performed, and empirical antibiotics were administered if the wound secreted purulent fluid. No bacterial specimen could be identified in either case. Patients with acute heart failure received interdisciplinary care from cardiologists. Patients with infections such as pneumonia or urinary tract infections received intravenous antibiotics. Patients with ileus received special attention for fluid and pain management. Two patients did not experience neurological improvement after surgery and reported increased pain. Postoperative CT revealed small epidural bleeding without significant compression, and surgery was not performed. Both patients’ condition improved 2 days after surgery, and pain levels were significantly reduced.

The most prevalent complications were pneumonia (20.0%) and urinary tract infection (8.6%). Detailed data regarding postoperative complications are presented in Table 3. In a secondary analysis, we examined potential risk factors for mortality. No significant association was found between postoperative bleeding and preoperative use of anticoagulants (OR 2.1 95% CI 1.1–2.7; *p*=0674). Comorbidities and postoperative complications were unique risk factors for mortality, whereas preoperative MS, blood transfusion, surgical duration, extension of surgery, and hospital or ICU stay were not (Table 4).

**Table 3:** Occurrence of adverse events

	Decompression *N*^a^ = 35
Deep wound infection	2 (5.7)
Acute respiratory failure	1 (2.9)
Acute heart failure	1 (2.9)
Pulmonary embolism	1 (2.9)
Pneumonia	7 (20.0)
Pleural effusion	2 (5.7)
Ileus	1 (2.9)
Urinary tract infection	3 (8.6)

All data are presented as the number of patients (%)

^*^Significant difference

^a^Group size

**Table 4:** Risk factors associated with mortality

Risk factor	OR (95% CI)	*p*-value
Age-adjusted CCI score	1.4 (1.1–3.5)	**0.001**
Preoperative MS	1.0 (0.9–1.1)	0.610
Duration of surgery	1.1 (0.9–1.3)	0.766
Number of levels decompressed	1.2 (1.0–2.3)	0.816
Blood transfusion	0.8 (0.7–1.2)	0.821
Length of ICU stay	1.3 (1.1–2.1)	0.281
Length of hospital stay	1.1 (1.0–1.7)	0.504
Complications	1.3 (1.2–3.4)	**0.004**

Bold *p*-values indicate statistically significant findings

*CCI*, Charlson comorbidity index; *CI*, confidence interval; *ICU*, intensive care unit; *MS*, motor score of the American Spinal Injury Association grading system; *OR*, odds ratio

## Discussion

A striking feature of the elderly is that they are prone to suffer a major spine injury from a seemingly minor trauma, such as a fall from standing or seating height. OF is a relatively common injury across the spinal cord in this age group, and biomechanical attrition is associated with osteoporosis and degenerative processes. Therefore, spine surgeons are frequently confronted with the management of such patients, who are at a higher risk of morbidity and mortality, mainly attributable to their associated underlying diseases ([25]. Nevertheless, the incidence of OF in the elderly has been predominantly mentioned as a byproduct in previous studies [33]), and a clear consensus on how to safely treat such debilitating patients is still debatable.

To the best of our knowledge, this is the first systematic analysis examining the clinical course of posterior screw fixation exclusively in octogenarians sustaining thoracolumbar or lumbar fractures due to minor trauma. We assessed morbidity and mortality rates and determined potential risk factors for mortality among octogenarians undergoing posterior instrumentation less than 72 h after trauma. We found that our patients had a very poor baseline reserve, as indicated by the CCI (mean 9.4), with cardiovascular diseases being the most prevalent. Notably, patients presented with higher grades of motor weakness (MS 84.1), whereas surgery led to significant amelioration of motor deficits. Notwithstanding, the in-hospital mortality was relatively high at 8.9%, while 90 days mortality was substantially lower at 2.9%. The occurrence of death was mainly attributable to postoperative complications, not surgery-related complications, with pneumonia presenting with the highest prevalence among the unsolicited events. Herein, it is important to highlight that patients’ baseline history and the occurrence of postoperative complications were unique risk factors for mortality, whereas the duration or extent of surgery was not.

### Review of literature

It is well known that spine surgery is associated with high-risk surgeries among older patients compared with their younger counterparts [11, 30, 32, 38]. A major explanation is the presence of multiple comorbidities and reduced functional reserves; thus, the management of OF is formidable when there is an urgent need for a surgical procedure. In their retrospective analysis of 47 octogenarians undergoing spinal surgery due to different spinal pathologies, this subset of patients predominantly suffered from cardiovascular diseases (appr. 97.0%), whereas 34% of the examined cohort presented with more than one comorbidity [32]. Notably, the results of regression analysis confirmed that multiple comorbidities were a significant predictor of mortality in octogenarians [32]. In line with these findings, a recent review and meta-analysis of mortality rates and associated risk factors after traumatic thoracolumbar vertebral fractures showed that both increasing age and the presence of underlying conditions are significantly associated with higher mortality rates [5]. The findings of another retrospective study based on claims data of 1979 older patients suffering from osteoporotic vertebral fractures with a mean age of 74 years pinpointed that despite age, an ASA score > 2 indicating the presence of a severe systemic disease constitutes a significant risk factor for higher rates of postoperative mortality [16]. Our study corroborates with the findings of the aforementioned studies. The distinct difference of our study is that we investigated only octogenarians with thoracolumbar and lumbar OF undergoing posterior screw fixation due to instability. Interestingly, we showed that all patients had a poor baseline history, which was a significant risk factor for postoperative mortality.

One might have deemed that such surgery might also contribute to mortality rates. It might seem surprising, but according to our findings, surgical type, duration, or extent of surgery did not have any significant impact on mortality rates. However, careful patient counseling is mandatory.

In the current study, the in-hospital mortality was relatively high at 8.6%, while the mortality rate at 90 days after surgery was 11.4%. Herein, it should be emphasized that the reported deaths were not surgery-related but were caused by acute heart failure or septic pneumonia. Consistent risk factors mainly explaining the occurrence of deaths were, as previously mentioned, patients’ comorbidities and also equally important the occurrence of postoperative complications. Considering these points, one might argue that refraining from surgery and following conservative management of the OF might be a deterrent to death. In their analysis of risk factors for mortality in octogenarians with spinal pathologies undergoing surgery, untreated vertebral fractures were found to be a significant predictor of mortality in this frail cohort [32]. In conjunction with these findings, Winkler et al. performed a retrospective analysis of 22.385 patients with thoracolumbar fractures based on claim data and advocated that instrumentation for thoracolumbar fractures significantly diminished the mortality rates, while surgery was associated with higher rates of perioperative complications [36]. Patients aged ≥ 70 years are at an increased risk of in-hospital mortality [36]. Similar to the results of the present study, the reported death rate for the elderly patients was 9.3%. Akin to Winkler et al., patients undergoing surgery for VF had significantly decreased risks for death, and on a long-term follow-up, surgically treated patients had lower mortality than those undergoing conservative management [13]. However, these results should be interpreted with caution because patients received only kyphoplasty, which is a brisk procedure with low intra- and perioperative risks, whereas spinal instrumentation can cause severe adverse events or even the need for revision surgery due to implant failure. Despite this notion, the findings of the current study ostensibly support that spinal instrumentation for the OF might be the key tool even for the treatment of such a debilitating cohort and even lead to better neurological outcomes, thus preserving patients’ quality of life. However, the occurrence of complications, such as pneumonia, should not be underestimated because they can lead to hazardous events and even death. In their study on perioperative and postoperative complications in patients undergoing either conservative or surgical management, Winkler et al. found higher odds for the occurrence of postoperative pneumonia and urinary tract infection in the elderly than in younger patients [36]. Concerning treatment modality, no substantial differences were observed between the groups [36]. Gupta et. al. reported similar complication rates in older patients undergoing surgery for OF, with pneumonia being the most prevalent [16]. Notably, in another study directly comparing kyphoplasty vs. posterior instrumentation in 25 patients with OF over 50 years of age, no significant differences were observed in morbidity and mortality among the groups; urinary tract infection was the most prevalent complication postsurgery [29].

The overall complication rates in the current study seem substantially higher (50.0%) compared to the above-mentioned studies. However, major difference between the studies is that we investigated only patients aged ≥ 80 years with a very poor baseline history; thus, after a surgical procedure, they are presumably amenable to the occurrence of adverse events compared with their younger counterparts. Nevertheless, on the one hand, the mortality rates are comparable to previous studies mainly examining younger patients [11, 32, 36]; however, on the other hand, posterior screw instrumentation results in significant improvements in the neurological status and preservation of patient functionality in daily activities. Because of this, we feel that our findings indicate that spinal instrumentation for thoracolumbar and lumbar OF might be the state of the art even for these individuals with such multitude needs.

Considering the potential complications after surgery, postoperative intensive care management might be essential to preserve or avoid reactions to complications that might occur. According to our results, all patients were postoperatively transferred to the ICU to achieve closer attention and better management of any potential complications due to the complexity of OF in combination with their additional history of comorbidities. Overall hospital stay was relatively long, with a duration of almost 13 days. Assuredly, it should be noted that ICU utilization is associated with high costs and a high demand for resources. However, such care seems to be necessary to keep morbidity and mortality rates as low as possible. Therefore, these findings are not surprising, since older patients warrant substantially more time to recover after general anesthesia due to the amenability to postsurgical complications, as previously described [26]. For instance, Pfeifle et al. reported on 260 older patients with a mean age of 78 years with acute OF and found a comparable hospital stay (16 days) to ours [26].

The surgical management of thoracolumbar OFs varies based on several factors, such as patient neurological status, fracture morphology, and surgeon preference. In this study, a surgical procedure was deemed necessary due to the fracture morphology and the presence of new acute neurological deterioration, despite the frailty of the cohort. The final decision on whether to use short- or long-segment posterior instrumentation was mainly guided by surgeon preference and the patient’s baseline medical history. There is an ongoing debate about whether to perform short- or long-segment instrumentation. Short-segment posterior instrumentation, as a standalone treatment, is a safe and effective modality in the management of thoracolumbar junctional injuries. Davis et al. conducted a retrospective study on 65 patients with unstable thoracolumbar fractures who underwent surgery and found that short-segment instrumentation provided a safe and effective treatment option, preserving or even correcting kyphotic deformity of almost 11° with very low implant failure rates [9]. In line with these findings, Liao et al. conducted biomechanical studies and found that in the case of OF burst fractures, short-segment posterior instrumentation with the additional insertion of cement-augmented short pedicle screws in the fracture vertebrae resulted in a strong construct, reducing stress at both upper and lower vertebrae [21, 22]. Another study reported satisfactory outcomes for patients with burst fractures of the thoracolumbar and lumbar spine who underwent surgical treatment with short-segment fixation [34]. However, for severe fractures with complete spinal injury or multiple fractures, long-segment posterior instrumentation is recommended as the state-of-the-art approach, although it sacrifices the motion of the fused spine and limits patient mobility [8]. Considering the aforementioned points and the poor baseline history of octogenarians, it appears that short-segment posterior instrumentation can lead to substantially good clinical outcomes in most cases and support swift patient mobilization while preserving against complications such as thromboembolic events attributable to longer immobilization times.

Osteoporotic fractures are a significant health concern for the elderly population, often resulting in severe complications. The primary cause of these fractures is the loss of mechanical resistance in the vertebrae under load. Due to changes in the vertebral micro-architecture, the thoracolumbar area experiences an increasing load as the transition from a stiff area to a mobile one occurs, along with changes in the native spine curves. Trauma, such as falling from sitting or standing, transfers compressive forces from the intervertebral disc to the vertebral plate and then to the vertebral body, leading to fractures. The loss of bone mineral density in older adults can further exacerbate these biomechanical changes, leading to the development of fractures [8, 21, 22]. Currently, there is no consensus on how to treat these fractures, especially when it comes to age-specific therapeutic concepts. The surgical approach typically aims to restore sagittal balance and sufficiently decompress the spinal canal, and the specific approach taken may vary depending on the institution and surgeon’s experience. Generally, long-segment instrumentation is recommended for patients with fracture dislocations, severe displacement, multiple fractures, or complete spinal cord injury, while short-segment fixation is indicated for patients with burst fractures [8]. However, it is important to note that there are distinct differences between young and old patients, especially in terms of bone quality. Due to the aging process, bone mineral density substantially decreases in older patients, making cement-augmented screws a more commonly utilized option for implant stability [20]. Additionally, older patients are more susceptible to postoperative complications, making less aggressive surgical procedures with shorter surgical durations a key tool in minimizing postoperative morbidity.

According to the spinal section of the DGOU, OF types 4 and 5 warrant dorsal instrumentation with or without vertebral body replacement or cement-augmented screws [27]. In concert with these guidelines, a posterior approach through short-segment fixation was performed to mitigate potential postoperative complications. It is also important to note that a point-to-point CT navigation for the insertion of the pedicle screws was used [17]; thus, not only providing maximal accuracy for screw placement, but also diminishing blood loss and facilitating swift surgery. Interestingly, only 2 patients underwent revision surgery because of wound infection, but none of the patients presented with screw loosening or secondary instability over a 3-year follow-up. In concordance with these findings, Lin et al., in their retrospective series on elderly patients with thoracolumbar OF, showed beneficial outcomes for both neurological status and spinal stability by performing short posterior screw instrumentation. Akin to Lin et al., our main goal was not to correct the sagittal alignment, but to improve the neurological status of the patients and to ensure the stability of the spinal cord. Although the occurrence of adjacent degenerative disease is not fully understood yet, and previous studies suggest that older patients and the length of the fusion (> 3 levels) are significant risk factors for its occurrence [7, 23], we also feel that the absence of ASD in these case series of very old patients is mainly attributable to the performance of short instrumentations. However, the optimal sagittal alignment in elderly patients with osteoporosis is debatable, and long-term follow-up focusing on residual kyphosis is necessary.

### Limitations

The main strength of the current study is that it is the first to examine such a large cohort of octogenarians undergoing instrumentation for thoracolumbar and lumbar fractures. However, this study has some limitations. Selection bias cannot be ruled out because of the retrospective study design. Additionally, as the data originated from a high-volume center, the potential performance bias should also be considered. A longer follow-up period may have uncovered other relevant information that was not captured in the current study. Special assessments for evaluating the degree of osteoporosis could not be performed preoperatively due to the emergent nature of surgical therapy.

During hospitalization, aggressive treatment for osteoporosis was not performed as the focus was on the emergency surgical management of fractures causing neurological deterioration in patients. However, an endocrinologist appointment was recommended for specific examinations and initiation of medical therapy. Due to the retrospective study design, we used the ASIA motor score to determine the motor status of patients, which may not fully capture the nuances of patients’ neurological condition. While a detailed description of patients’ neurological condition might provide more insight into the pathology, we believe that the use of the ASIA motor score provided substantial information on patients’ clinical status and allowed the investigation of potential associations between neurological status and patient outcomes. Finally, the lack of a conservatively treated control group might have been an additional limitation.

## Conclusions

The findings of the present study confirm that pedicle screw fixation for thoracolumbar and lumbar OF in octogenarians led to significant improvement in neurological status. Mortality was associated with poor baseline reserve and postoperative complications. Therefore, we strongly believe that close postoperative monitoring is essential for patient clinical outcomes. Interestingly, no revision surgery due to screw loosening or secondary instability was necessary, indicating that short-segment fusion might be the key tool for such a pathology, even in octogenarians. Nevertheless, a clear discussion with the patient and relatives regarding the potential risks is unambiguously recommended.

## Data Availability

The datasets generated during and/or analyzed during the current study are available from the corresponding author on reasonable request.
